# Sub-clinical systolic dysfunction with persistent myocardial edema and inflammation in elite high-endurance athletes with common colds: a cardiovascular magnetic resonance study

**DOI:** 10.1186/1532-429X-11-S1-O3

**Published:** 2009-01-28

**Authors:** Myra S Cocker, Oliver Strohm, David J Smith, Craig Butler, Israel Belenkie, Willem Meeuwisse, Matthias G Friedrich

**Affiliations:** 1 Stephenson CMR Centre at the Libin Cardiovascular Institute, University of Calgary, Calgary, AB Canada; 2 Human Performance Lab, Faculty of Kinesiology, University of Calgary, Calgary, AB Canada; 3 Department of Cardiac Sciences at the Libin Cardiovascular Institute, University of Calgary, Calgary, AB Canada; 4 Sports Medicine Centre, University of Calgary, Calgary, AB Canada

**Keywords:** Influenza, Cardiovascular Magnetic Resonance, Myocardial Inflammation, Adverse Cardiac Event, Common Cold

## Background

Basic research has demonstrated that myocardial inflammation may be a feature of systemic viral inflammation, resulting from agents such as influenza. Physical activity during exposure to pathogens has been shown to exacerbate the propensity to develop adverse cardiac events. As such, based upon empirical findings, current guidelines on athletic training deter athletes from participating in sport during common colds. Cardio-vascular Magnetic Resonance (CMR) allows for non-invasive visualization of myocardial inflammation, where it has emerged as the imaging modality of choice to assess the course of myocarditis. Thus, using CMR-based tissue characterization, we hypothesized that colds in elite high-endurance athletes would lead to depressed cardiac function and myocardial inflammation.

## Methods

62 (32 male, 31 ± 13 years) elite high-endurance athletes were prospectively recruited. CMR scans were performed at baseline, with an acute common cold, and 4 weeks after. Pre-defined symptoms were used to rule in an acute cold. LV function, edema, and myocardial inflammation were assessed using standard SSFP, T2-, and T1-weighted imaging, respectively, on a 1.5 T MRI system.

Standard, previously described approaches for the quantification of LV function, edema and myocardial inflammation were utilized. Statistical comparisons were performed with repeated measures ANOVA, at 2 levels of measurement.

## Results

During the 11-month period of recruitment, 21 athletes completed all 3 scans. During an acute cold, we observed a significant increase in LVESVI, with reduced LVSVI and LVEF (p < 0.05), while LVEDVI and LVMI did not differ (Table 1). Moreover, there were no statistical differences between LV volumes at the 4-week follow-up to those at baseline or with an acute cold.

**Table 1:** LV volume and CMR markers for edema and inflammation at baseline, with a common cold and at a 4-week follow-up. Volumetric data are presented as mean standard deviation

	Baseline visit	Visit with common cold	4-week follow-up
LVEDVI (ml/m)	111.4 ± 20	110.0 ± 22	109.9 ± 21
LVESVI (ml/m)	39.4 ± 11	41.7 ± 11*	40.6 ± 9
LVSVI (ml/m)	72.0 ± 12	68.3 ± 13*	69.3 ± 15
LVEF (%)	65.0 ± 4.8	62.5 ± 4.9*	63.0 ± 5.8
LVMI (g/m)	58.8 ± 15	59.1 ± 15	60.1 ± 16
Edema (n)	4 of 21	4 of 21	5 of 21
Inflammation (n)	7 of 21	8 of 21	10 of 21

*p < 0.05 baseline compared to visit with common cold.

**Figure 1:**
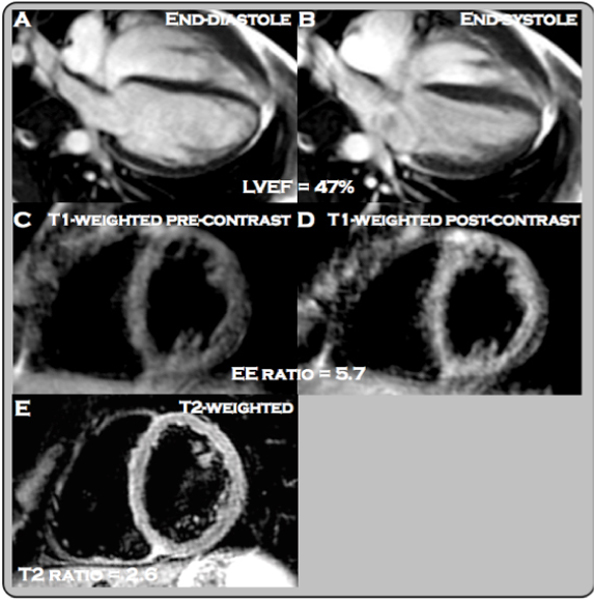
**Reduced contractile function in an elite professional swimmer with a common cold (LVEF**_**cold**_ **47%, LVEF**_**baseline**_ **54%) (A, B)**. Visually apparent increased early enhancement post-contrast in T1-weighted images (**C**, **D**) suggestive of myocardial inflammation, with an early enhancement ratio of 5.7. Evidence of global myocardial edema (**E**, ratio 2.6).

In terms of tissue characterization, 19% of athletes had evidence for myocardial edema with an acute cold, and 24% at follow-up (Figure 1). 38% had myocardial inflammation during an acute cold; and this proportion increased to 48% at follow-up.

## Conclusion

We provide first evidence of sub-clinical myocardial involvement with common colds in high-endurance athletes. Colds were associated with a small yet significant decrease of systolic function, and persisting myocardial inflammation visualized with CMR-derived markers for edema and inflammation. Further research is required to investigate the implications of these findings on athletic performance.

